# Hand grip strength, vitamin D status, and diets as predictors of bone health in 6–12 years old school children

**DOI:** 10.1186/s12891-023-06960-3

**Published:** 2023-10-23

**Authors:** Ahmad H. Alghadir, Sami A. Gabr, Amir Iqbal

**Affiliations:** Department of Rehabilitation Sciences, College of Applied Medical Sciences, King Saud University, P.O. Box 10219, Riyadh, 11433 Saudi Arabia

**Keywords:** Vitamin D status, Hand grip strength, Physical activity, School children, BMD, Adiposity

## Abstract

**Background:**

Vitamin D and calcium-rich foods, exposure to sunlight, and physical activities (PA) play a pivotal role in promoting the production of sufficient vitamin D and improving grip strength needed for better bone health among school children.

**Purpose:**

This study aimed to determine the effects of hand grip muscle strength (HGS), vitamin D in addition to diets, and PA on bone health status among 6–12 years old schoolchildren.

**Methods:**

This study was based on a cross-sectional observational design, which was descriptive in nature. A diverse sample of 560 elementary school children aged 6–12 years old were invited to participate in this descriptive cross-sectional study. The Dual-Energy X-Ray Absorptiometry (DXA), QUS technique, and ACTi graph GT1M accelerometer were used respectively as a valid tools to identify BMD, BMC, and other parameters of bone health like c-BUA values and bone stiffness (SI), and physical activity (PA) of all individuals participated in this study. In addition, a hydraulic dynamometer was used to measure hand grip strength among the participants. Moreover, an immunoassay technique was used to measure the serum levels of vitamin 25(OH)D level, and bone metabolism markers; NTX, DPD, Ca, and sBAP in all participants. Bone loss (osteoporosis) was cross-sectionally predicted in 19.64% of the total population, most of whom were girls (14.3% vs. 5.4% for boys; *P* = 0.01). Compared to boys, the incidence of osteoporosis was higher and significantly correlated in girls with lower HGS, deficient vitamin D, inadequate vitamin D and Ca intake, greater adiposity, poor PA, and lower sun exposure. Also, in girls, lower vitamin 25(OH)D levels, and poor HGS were shown to be significantly associated with lower values of BMD, BMC, SI, and higher values of bone resorption markers; NTX, DPD, and sBAP and lower serum Ca than do in boys. The findings suggested that deficient vitamin D, lower HGS, adiposity, PA, and sun exposure as related risk factors to the pravelence of bone loss among school children, particularly in girls. In addition, these parameters might be considered diagnostic non-invasive predictors of bone health for clinical use in epidemiological contexts; however, more studies are required.

## Introduction

Skeletal muscles are one of the main forces that can generate a maximum enormous reaction during human activities [1–3]. These muscle forces produce a trophic or adaptive effect on bone mass locally during exercise training. Thus, a satisfied skeletal muscle adaptation leads in such a way to an increase in humeral bone mass [1–3]. In addition, hand grip strength as a model of muscle adaptation technique showed a positive association with local bone mass at the wrist or forearm among nonathletes [4–18]. Previously, grip strength showed a positive significant association with bone mass at other sites, including the hip and/or spine in females however, insignificant association was reported between grip strength and bone mineral density (BMD) among men [17–19]. The grip strength is associated with the incidence of osteoporotic fractures, particularly vertebral fractures [20]. In recent studies, grip strength exercises was used as a treatment strategy for the patients with primary subacromial impingement syndrome [21]. Also, it was report in recnt study that grip strength along with the levels of vitamin D might be used as a pridector of liver fibrosis and malnutrition in patients with Chronic Hepatitis C virus (HCV) [22].

During childhood and adolescence, the growth of a healthy peak bone mass is needed for better bone health which might be a key limited factor of bone health and future fracture risk during adulthood [23, 24]. A number of physiological and lifestyle factors such as genetics, hormonal, both calcium and vitamin D intake, physical activity, and nutrition showed to be effective in the bone health of children and adolescents [25, 26]. Hand grip strength was associated with bone density and mineral content in children and adolescents [27–30]. In addition, other physiological factors such as maximum oxygen consumption and maximum expiratory flow were shown to be significantly associated with bone mineral density in children, adolescents, and adults [27–30].

Physical activity (PA) with higher levels showed to optimize skeletal development, and significantly aid to prevent age-related bone loss and osteoporotic fractures [31–35]. The correlation between muscle optimization and bone status was suggested to be mechanical in origin, whereas the skeletall system clearly adapts to both stress and mechanical loads on muscles which produce powerful loading forces on the bone [36]. In school-based interventions, high-impact PA was clearly observed to improve both the muscle force and strength acting on the bone [37, 38]. These observations obviously suggested the pivotal role of, muscle strength and muscle mass in bone development during growth [39]. Conversely, children with sedentary life behaviors, such as sitting for a long time in front of screens watching television or playing computer games may have a negative or adverse effect on bone health [31, 40–43].

Vitamin D also showed to have a prospective role in skeletal muscle structure, function, and bone health [44, 45]. Muscle weakness and poor bone health were shown to be linked with vitamin D deficiency in both children and adults. This significantly supports the indirect benefits role of vitamin D on skeletal health via the regulation of calcium homeostasis [43]. Previous reports confirmed the role of vitamin D in muscle function, calcium absorption, and maintaining homeostasis [44–55]. In cases with vitamin D deficiency, poor muscle function appears as a sign of bone disease before the biochemical bone profiles [46, 47].

In schoolchildren, serum 25(OH)D levels showed to be associated with handgrip strength. In these studies, the authors signify the pivotal roles of vitamin D levels, calcium and vitamin D intake, and exposure to sunlight in improving hand grip strength and bone health [48].

Given the potential positive impact of vitamin D status and diets on both muscle strength and bone loss risks, it is important to consider the role of vitamin D and its relationship between muscle strength and bone health among school children [41–48]. Thus, the proposed aim of this study was to evaluate the effects of hand grip muscle strength, vitamin D in addition to diets, sunexposure, and physical activity on bone health status among a diverse sample of schoolchildren aged 6–12 years old.

## Materials and methods

### Study participants

This descriptive cross-sectional study included a total of five-hundred sixty elementary schoolchildren (boys *n* = 360 and girls *n* = 200) aged 6–12 years old who were invited via emails to the respective administrative personnel during September 2018 and May 2019. The required students were Students with physical, genetic, endocrine, and cardiovascular disorders who were prevented from participating in this study. Also, students with chronic diseases, including diabetes, cardiac, pulmonary, and neurological diseases as well as acute infections, or who received medical therapy or diets that may affect on the data of vitamin D and bone were excluded from this study [49].

### Study settings

The sample size of 560 was selected from the list of students in 10 public schools in a large geographical area of Riyadh province to give an estimated power of 96% and a significance level of 0.05 with an expected frequency of 5.4%. The study was performed at CAMS, King Saud University, and supervised by an expert physiotherapist with more than 10 years of experience. Blood samples were collected from all subjects using a heparinized syringe, and plasma samples were obtained from whole blood following centrifugation for 1 min at 1400 rpm. The samples were frozen at 20° C until use [49]. Demographic and clinical data of the participants are in Table 1.

**Table 1:** Baseline characteristics of clinical, laboratory, physical activity and diet scores for children aged 6–12 years based on hand grip strength (*n* = 560; mean ± SD)

Variables	Grip strength scores			*P*-value
	Low (HGS; 0–230mmHg)	Normal(HGS ≥ 300mmHg)	Moderate (HGS;231–299mmHg)	
No	100	300	160	-
Age (years)	10.5 ± 1.2	10.2 ± 1.5	10.5 ± 1.3	0.145
Gender (boys/girls)	38/62	185/115	70/90	-
Anthropometry				0.001
BMI	26.9 ± 4.8	21.6 ± 2.3	29.6 ± 6.7	
WHtR	0.85 ± 0.13	0.45 ± 0.08	1.2 ± 0.16	
Body composition (DXA):				0.001
Percentage of Fat (% G)	26 ± 2.9	21.9 ± 3.2	31.5 ± 6.4	
Fat Mass (kg)	22.3 ± 1.5	18.5 ± 4.2	25 ± 6.1	
Lean Mass (kg)	22.9 ± 2.7	48.3 ± 4.8	26.49 ± 9.3	
Bone Health				0.001
BMD (g/cm2) *Body total:*	0.8 ± 0.12	1.3 ± 0.18	0.96 ± 0.10	
BMC (g) *Body total:*	1.98 ± 0.48	2.63 ± 0.58	2.1 ± 0.62	
Bone stiffness index (SI)	62.4 ± 3.96	118.4 ± 12.3	78.4 ± 11.8	
Isometric strength (lbf)				0.001
Right Hand	186 ± 10.8	356 ± 12.5	275 ± 11.2	
Left Hand	125 ± 7.8	317 ± 8.9	248 ± 8.6	
Diabetes				0.131
FBG (mmol/L)	4.1 ± 0.26	3.8 ± 0.31	3.9 ± 0.45	
HbA1c	3.4 ± 1.2	2.8 ± 0.81	3.1 ± 0.61	
Diet Measurements				0.001
Diet score	16.8 ± 4.1	32.6 ± 2.8	21.7 ± 3.7	
Dietary vitamin D intake (IU/d)	136 ± 62	196 ± 82	148 ± 71	
Dietary Ca intake (mg/d)	680 ± 115	1200 ± 168	896 ± 120	
Sun exposur (h/day)	1.7 ± 0.9	3.9 ± 1.5	2.6 ± 1.1	0.001
PA				0.001
Total PA (counts/min)	480 ± 128	1320 ± 250	816 ± 168	
MVPA (%)	17.6	78.6	31.5	
Total energy (kcal/d)	1260 ± 318	4975 ± 618	2348 ± 456	

All values were reported as mean ± SD or median (interquartile range) or percentage. Kruskal–Wallis one-way ANOVA, and post-hoc (Tukey HSD) test were used to compare the mean values of the studied variables. Variables were considered significantly different at *P* < 0.05

*Abbreviations*: *FBG* Fasting blood sugar, *HbA1C* Glycated hemoglobin A1c, *BMI* Body mass index, *WHtR* Waist to height ratio, *MVPA* Moderate-to-vigorous physical activity, *PA* Physical activity, *BMD* Bone Mineral Density, *BMC* Bone Mineral Content, *HGS* Hand grip strength

### Ethical consideration

Based on the ethical guidelines of the 1975 Declaration of Helsinki, the study protocol was reviewed and approved by the ethics Sub-Committee of King Saud University, Saudi Arabia. Assignment of a signed written informed consent was obtained from the parents of all participating schoolchildren before collecting data and blood samples of each participant.

### Anthropometric measurements

Standardized procedures such as a tape measure and calibrated Salter Electronic Scales (Digital Pearson Scale; ADAM Equipment Inc., Columbia, MD, USA) were used to measure the height and weight of all participants, respectively. Adiposity parameters such as BMI and Waist-to-height ratio (WHtR) were calculated according to previously validated universal cutoff values [45–48]. These universal WHtR cut-off values were based on data obtained from schoolchildren internationally and were significantly appreciated to identify severe complications, particularly early cardiovascular risk in both children and adolescents [50–53].

### Assessment of bone structure and body composition

All participants were subjected to a total body scan by using “the Dual-Energy X-Ray Absorptiometry (DXA) (Lunar Prodigy; General Electric, Fairfield, CT) as previously reported in the literature [54]. According to the manufacturer’s instructions, all measurements were evaluated by a well well-trained technician. For each participant, both the BMD (g/cm2) and the BMC (g) were measured in addition to other body composition variables, such as the percentage of body fat (% F), muscle mass (kg), bone mass (kg), and fat mass (kg) respectively. To estimate the reliability of the scan, the measurements were repeated directly on the same day to obtain lower technical error of measurement (TEM; < 1..5%) as previously estimated [54, 55]. T-scores are a commonly used method for assessing bone health in both children and adults. T-scores are calculated by comparing an individual’s bone mineral density (BMD) to the average BMD of a healthy young adult of the same sex. This comparison is made using a statistical measure known as a standard deviation (SD), which expresses how much an individual’s BMD differs from the average young adult BMD [56, 57]. In children, T-scores are often used to identify bone health because they provide a way to compare a child’s BMD to that of a healthy young adult, which is considered the “gold standard” for bone health [56–58]. This is important because children’s bones are still growing and developing, and their BMD can vary widely depending on their age, sex, and other factors such as nutrition and physical activity. Thus, in terms of accuracy, T-scores are generally considered the most reliable measure of bone health in children and adults. So, according to the T-score obtained from the DXA scan measurements, osteoporosis was diagnosed among participants as normal (T-score ≥ −1), osteopenia (low bone density; T-score; −1 to −2.5), osteoporosis (T-score ≤ −2.5), and severe or established osteoporosis (T-score; ≤  − 2.5 with fracture [58].

A commercially used Achilles ultrasound densitometer (Lunar Corporation, Madison, WI) QUS technique was used to measure bone health and stiffness (SI) of all participants, as reported previously [57, 58]. As previously reported, both c-BUA values and bone stiffness (SI) were significantly related to bone health in young individuals, [59, 60]. Based on broadband ultrasound attenuation (dB/MHz) and speed of sound (m/s) parameters, both c-BUA values and bone stiffness (SI) were identified in brief detail in young individuals on the left and right calcaneus as previously reported [61–64]. QUS measurements are correlated with DXA measurements and are used as a valid tool for indicating the risk of osteoporotic fractures in children [61, 62]. In addition, it shows a good reflection on bone changes during growth and recorded the strongest association with DXA measures of bone mass in children [65, 66].

In children, however, the clinical usefulness of QUS has not yet been investigated, and comparison studies showed inconsistent correlations with DXA [59, 60]. In clinical practice, poor bone health was considered among participants when c-BUA values were recorded below or equal to QUS- *Z*-score cutoff of ≤ −1.5 as previously mentioned [67], and that c-BUA values were coefficiently varied from 0.69% to 1.8% within-day of measurements as mentioned in the literature [68].

### Assessment of hand grip strength

For each participant, a manual hydraulic dynamometer labeled JAMAR (Hydraulic Hand Dynamometer® Model PC-5030 J1, Fred Sammons, Inc., Burr Ridge, IL: USA) was used to measure hand grip strength with 0.1 lbf accuracy of both the right and left hands as previously reported in the literature [55, 62]. First, in the standard position, each student was seated in a straight-backed chair. Then, he was asked to squeeze the dynamometer two times with each hand. For each hand, approximately 2-min rest lapsed between trials to control for the effects of fatigue on each hand alternated. The best value of two attempts was recorded. The inter-rater Technical Error of Measurement was less than 2.5% for both hands [55, 69, 70]. Based on grip strength scores (HGS), children were classified into three groups; Low (*n* = 100; HGS; 0–230mmHg), normal (*n* = 300; HGS ≥ 300mmHg), and moderate (*n* = 160; HGS;231–299mmHg) respectively [71–73].

### Diet information and physical activity

All schoolchildren were instructed not to change their normal eating habits during the study period. Parents were asked to record accurately the amount, type of food, and fluid consumed using food diaries. For each participant, dietary information was extensively referred according to reference dietary intakes for physically active people [74, 75].

Physical activity for each participant was evaluated for 7 consecutive days using ACTi graph GT1M accelerometer (model WAM 7164; Fort Walton Beach, FL). The average intensity of PA was calculated from the total number of minutes each child participated in sports activities with different intensities. This intensity is based mainly on count thresholds and daily activity counts per minute. Children with fewer accelerometer counts (≤ 100 counts/min) were characterized by a sedentary lifestyle [76, 77]. According to energy expenditure, the PA of all participants was classified as low or sedentary ( thresholds are less than 4 metabolic equivalents [METs], moderate activity (thresholds of 4 metabolic equivalents [METs]), and vigorous activity (thresholds of 7 METs), respectively as previously mentioned, whereas 1 MET refers to either energy expenditure of 1 kcal/kg/h or oxygen uptake in 3.5 mL/kg/min during a quiet sitting position [69, 78].

### Assessement of 25-hydroxyvitamin D and bone metabolism

From freshly separated serum samples of each student, serum vitamin 25(OH)D level, NTX, DPD, Ca and sBAP concentrations were estimated as outcome measures of bone health as previously reported [79–83]. Colorimetric and immunoenzymometic assays along with immunoassay kits such as (IDS, Tyne & Wear, UK) for vitamin 25(OH)D, (Hoffmann-La Roche Ltd., Basel, Switzerland) for Ca levels, and (Quidel Corporation, San Diego, CA, USA) for sBAP concentrations (U/L) were significantly used to measure Vitamin 25(OH)D levels, Ca levels, and sBAP concentrations (U/L in serum samples of the participating students as mentioned previously in the literature [79–83]. Also, ELISA kits (Osteomark, Ostex International, Seattle, WA, USA) for NTX and enzyme immunoassay kits (Metra Biosystems, Mountain View, CA, USA) for DPD were used to estimate the levels of both NTX and DPD respectively in urine samples of the participating students using enzyme immunoassay techniques [81].

Due to the importance of sunlight exposure is an important source of vitamin D synthesis which contributes to the bone mineralization process [84]. Thus, all schoolchildren’s daily exposure to sun during the previous month was estimated as the average number of hours per day the students were exposed to the sun [85, 86]. The mean daylight duration of exposure was adjusted to be (±0.1h) as previously calculated using astronomical tables [60].

### Statistical analysis

In this study, for the analysis of the data, the statistical software SPSS version 18 was used. The results obtained were expressed as Mean and standard deviation Among groups, Kruskal–Wallis one-way ANOVA, and post-hoc (Tukey HSD) test were used to compare the mean values of the studied variables [54]. The relationship between various study parameters were performed by spearman rank correlation analysis. Linear regression analysis was performed in steps for hand grip strength, bone markers, vitamin D, Ca intake, and lifiestyle paramters like physical activity (PA), sunexposure, and adiposity as the independent variables and BMD, BMC,BSI,and osteoprosis as dependent variables. The data obtained were deemed significant at *P* < 0.05 [54].

## Results

A total of 560 school children aged 6–12 years old were recruited in this descriptive cross-sectional study to evaluate the effect of hand grip strength (HGs), vitamin D, and dites on bone health. Based on hand grip strength measurements, children were classified into three groups; Low (*n* = 100; HGS; 0–230mmHg), normal (*n* = 300; HGS ≥ 300mmHg), and moderate (*n* = 160; HGS;231–299mmHg) respectively. All studied variables were described statistically as shown in Table 1.

**Fig. 1:**
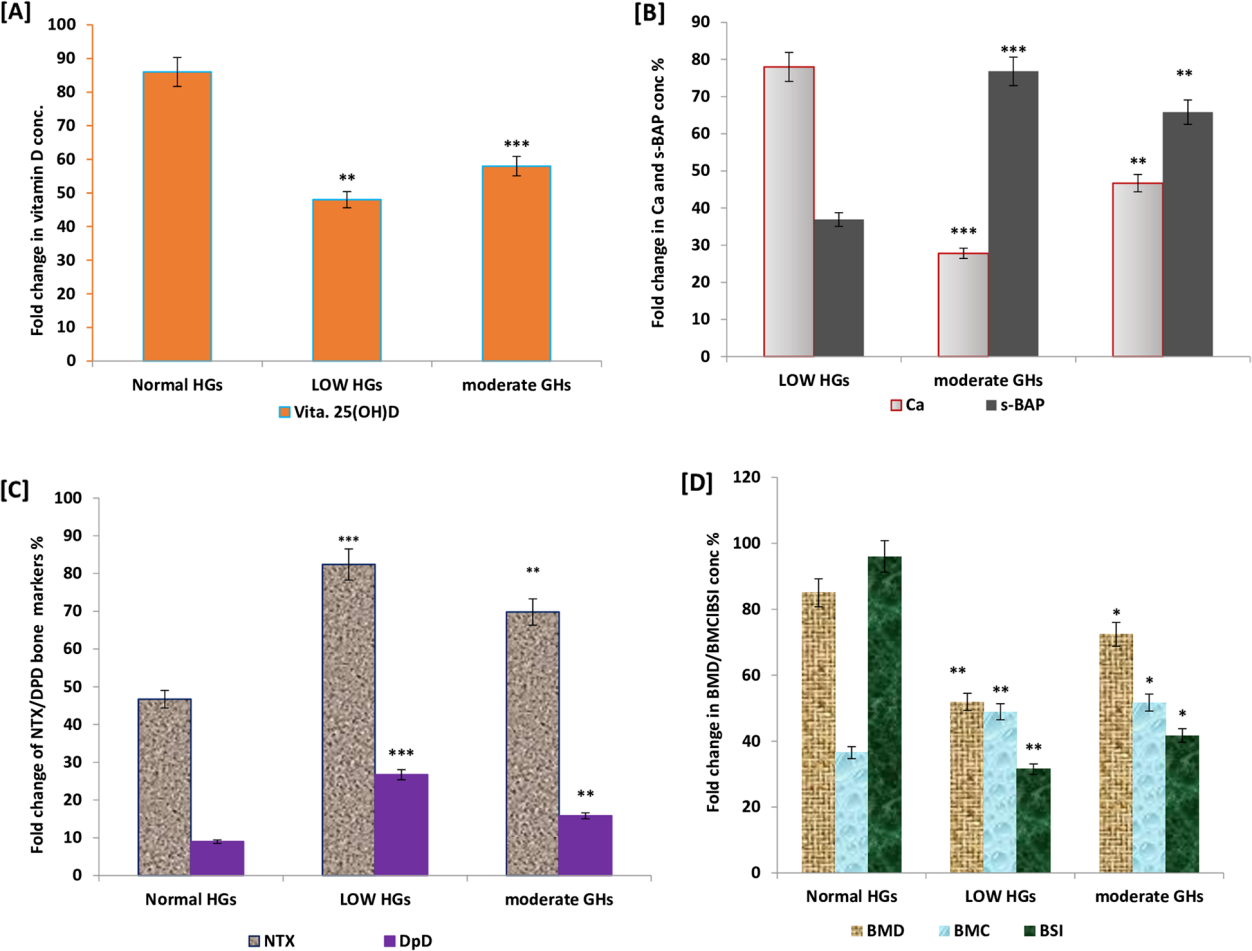
BMD, BMC, vitamin D, and realated bone marker values; NTX, DPD, s-Ca, s-BAP for school childern based on hand grip strength expressed in categories (low, normal and moderate). **A** Significant difference in vitamin D status in school children with low (*p* = 0.01) and moderate (*p* = 0.001) grip strength compared to those with normal HGS, **B** significant decerease (*p* = 0.0001) in serum Ca, and increase in s-BAP (*p* = 0.01) was estimated in subjects with lower to moderate HGS, **C** significant increase (*p* = 0.001; *P* = 0.01) in the levels of bone markers; NTX and DPD in subjects with lower HGS, **D** significant decrease in total BMD, BMC, and BSI index was estimated in all school chikldern with lower (*p* = 0.001) to moderate (*p* = 0.05) HGS respectively

In relation to children with normal HGs, children with lower and moderate HGs showed a greater percentage of fat, body fat, adiposity markers; BMI, WHtR, and lower percentage of lean mass, total body BMD, total body BMC, bone stiffness index (BSI), and isometric grip strength (right and left) respectively (*p* < 0.001) as shown in Table 1 and Fig. 1D. Also, lower diet scores, inadequate vitamin D and calcium intake, lower sun exposure, and lower physical activity were significantly reported in children with lower and moderate HGs compared to subjects with normal HGs (Table 1).

In this study, serum vitamin 25(OH)D level, NTX, DPD, Ca, and sBAP concentrations were estimated as outcome measures of bone health among studied children (Fig. 1). A significant decline in 25(OH)D levels was estimated in school children with low (*p* = 0.01) and moderate (*p* = 0.001) grip strength compared to those with normal HGS (Fig. 1A). Also, a significant decrease (*p* = 0.0001) in serum Ca, and an increase in s-BAP (*p* = 0.01) concentrations were estimated in subjects with lower to moderate HGS (Fig. 1B). Similarly, NTX and DPD as markers of bone resorption were significantly increased in children with lower (*p* = 0.001) and moderate HGs (*P* = 0.01) respectively in comparison with those of control subjects (Fig. 1C).

**Fig. 2:**
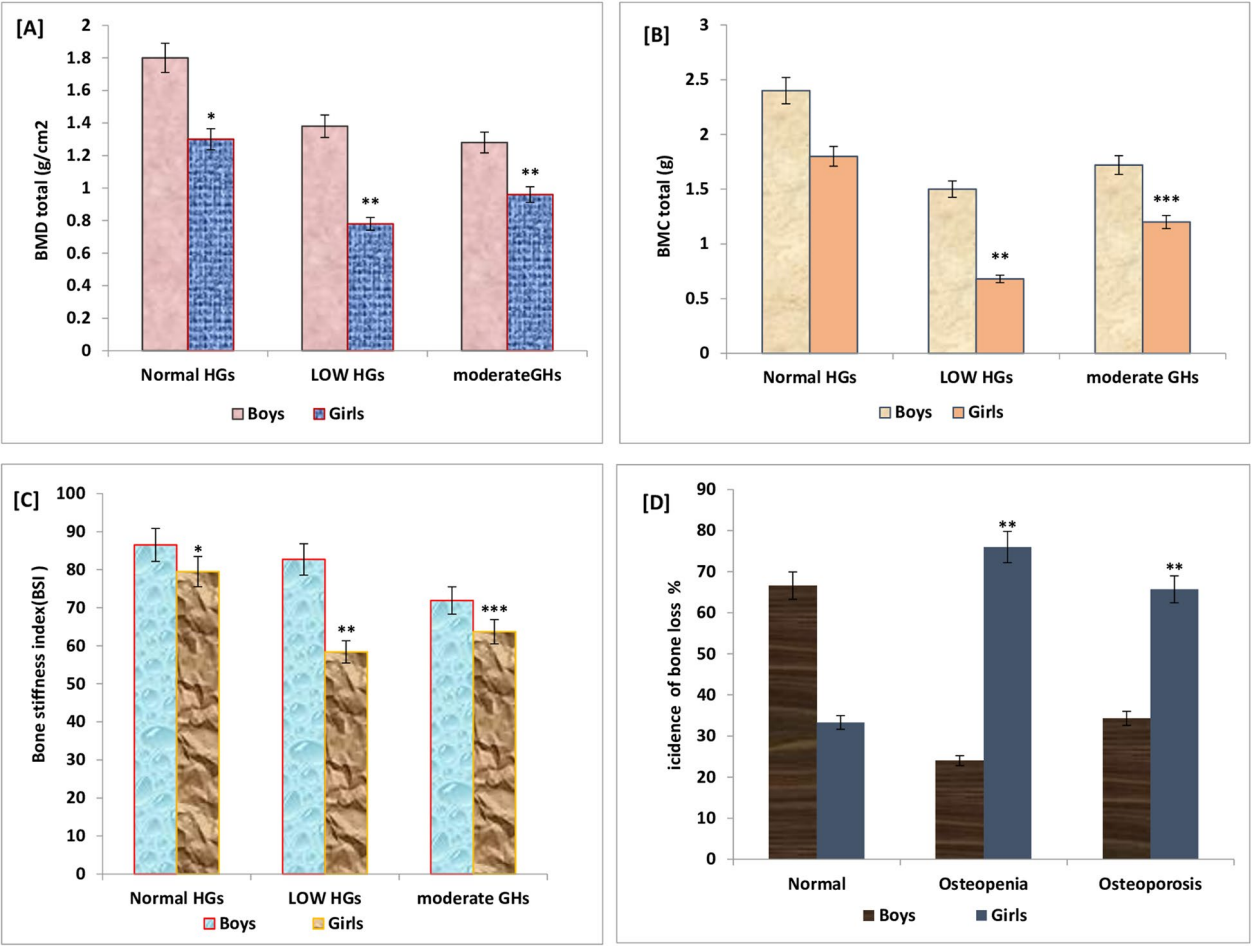
BMD, BMC, BSI, and bone loss values for school boys and girls based on hand grip strength expressed in categories (normal, moderate and low HGs). Significant decrease in BMD (**A**), BMC (**B**), and BSI (**C**) were estimated in school girls compared to boys. Also, bone loss or osteoporosis was significantly estimated in girls (*p* = 0.01) than in boys (**D**)

**Fig. 3:**
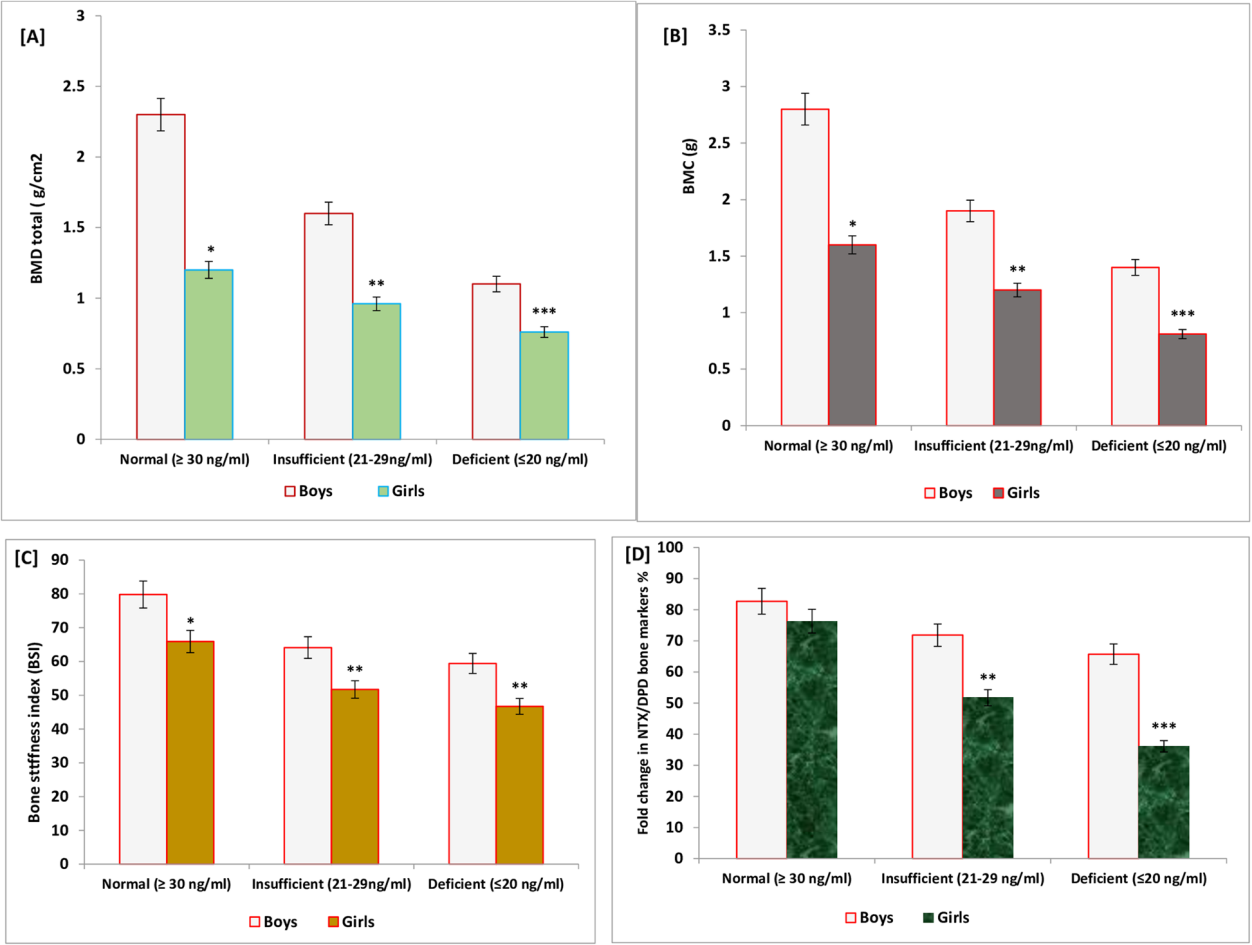
BMD, BMC, BSI, and realated bone marker values; NTX, DPD for school boys and girls based on vitamin D status expressed in categories (normal, insufficient, and deficient). Significant decrease in BMD (**A**), BMC (**B**), BSI (**C**) along with an increase in the levels of bone resorption markers NTX and DPD (**D**) were estimated in school girls compared to boys

Figures 2 and 3 illustrate the significant differences in BMD, BMC, BSI, and the percentage of bone loss or osteoporosis based on the categories of hand grip strength and vitamin D status. In both genders, differences occurred between the three categories of hand grip strength (HGS). Moreover, lower BMD, BMC, and BSI, (Fig. 2A, B, C) were significantly estimated (*p* = 0.01) in girls when compared to the boys in all the categories of HGS.

Also, in this study bone loss (osteopenia/ osteoporosis) was significantly predicted in 19.64% of the total populations most of them were girls (14.3% vs 5.4% for boys; *P* = 0.01). The incidence of osteoporosis was higher and significantly correlated in girls with lower and moderate HGS than in boys of the same category as shown in Fig. 2D. The data also showed that increases in obesity, lower HGS, deficient vitamin D status, diets containing inadequate vitamin D and Ca values, sedentary lifestyle or lower PA, and inadequate exposure to sunlight, in addition to higher expression of bone resorption markers were significantly considered as related risk factors to the prevalence of bone loss among school children particularly in girls (Table 2).

**Table 2:** Risk of bone loss in relation to grip strength, vitamin D status, markers of bone metabolism, gender, adiposity, diets, and physical activities among school children

Parameters	n	Bone loss prevalence (%)	OR (95% CI)	P-value
Gender				0.001
Boys	30	5.4	1.3 (0.86–1.6)	
Girls	80	14.3	1.9 (0.8–2.1)	
Adiposity				0.001
Obese (≥ 25)	260	46.43	1.86 (0.86–2.5)	
Non-obese (≤ 25)	300	53.57	0.96 (0.72–1.35)	
Hand grip strength				0.001
Low (0–230mmHg)	100	17.6	0.52 (0.26–1.3)	
Moderate (231–299mmHg)	160	28.6	0.86 (0.56–1.9)	
High (normal; ≥ 300mmHg)	300	53.6	1.74 (0.76–2.3)	
Vitamin D status				0.001
Normal (≥ 30ng/ml)	300	53.6	1.7 (0.78–1.9)	
Insufficient (21–29 ng/ml)	160	28.6	0.85 (0.36–1.6)	
Deficient (≤ 20 ng/ml)	100	17.6	0.56 (0.48–1.3)	
Vitamin D and Ca diets				0.001
Adequate	300	53.6	2.3 (0.86–2.6)	
In adequate	260	46.4	0.84 (0.46–1.2)	
Bone markers				0.001
Normal	300	53.6	1.68 (0.66–2.1)	
High	260	46.6	0.76 (0.56–1.7)	
Physical activity				0.001
Active	300	53.6	1.8 (0.75–2.6)	
In active	260	46.6	1.1 (0.86–1.6)	
Sun exposure ((h/day)				0.001
Adequate	300	53.6	1.8 (0.75–2.6)	
Inadequate	260	46.6	1.1 (0.86–1.6)	

*N* = 560; data are odds ratios from logistic regression models; *P*-value < 0.05. ORs adjusted for age and gender in a logistic regression model

*OR* Odds ratio, *CI* Confidence interval

The correlation between vitamin D status and bone health was evaluated in this study. A significant decrease in BMD, BMC, and BSI (Fig. 3A, B, C) along with an increase in the levels of bone resorption markers NTX and DPD (Fig. 3D) was estimated in school girls who showed a deficient (*P* = 0.001) or insufficient (*P* = 0.01) vitamin D status compared to boys of the same category.

In this study, linear regression analysis in step multiples revealed that left and right hand grip strength, vitamin 25(OH)D status, bone markers; NTX, DPD, Ca,s-BAP, diet scores, adiposity, PA, and sun exposure variables were associated with the bone health (BMD, BMC, and BSI), and incidence rates of osteoporosis among school children of both genders. The percentage of explained variation was greater in boys than girls as shown in Table 3. For example; left and right hand grip for boys showed *R* = 0.76 *R*2 = 0.48 with percentage of 76% and 48% with higher estimate of 10% than for girls *R* = 0.58, *R*2 = 0.29 with EE = 6% compared to boys. Thus, in our Table 3, the results are more than zero with a ratio percentage higher than estimated for girls and were concluded that the selected cofounders could predict the health of bones among the selected schoolchildren.

**Table 3:** Estimation of the BMD, BMC, BSI predictors, and osteoporosis based on linear multiple regression of school children

Dependent variables	Independent variables	Boys			Girls				
		*R*	*R*^2^	EE	*P*	*R*	*R*^2^	EE	*P*
BMD total (g/cm2)	Hand grip strength(left/right)	0.76	0.48	0.10	0.001	0.58	0.29	0.06	0.001
	Vitamin 25(OH)D status	0.61	0.36	0.13	0.001	0.37	0.18	0.09	0.001
	Bone markers (NTX,DPD, Ca,s-BAP)	0.79	0.42	0.11	0.001	0.46	0.16	0.09	0.001
	Vitamin D and Ca intake	0.65	0.38	0.16	0.001	0.52	0.11	0.13	0.01
	physical activity/sun exposure	0.60	0.48	0.28	0.001	0.35	0.21	0.19	0.01
	Adiposity (BMI, WHtR)	0.76	0.18	0.36	0.001	0.58	0.12	0.27	0.01
BMC total (g)	Hand grip strength(left/right)	0.81	0.69	0.32	0.001	0.65	0.26	0.21	0.001
	Vitamin 25(OH)D status	0.56	0.26	0.27	0.001	0.42	0.16	0.22	0.001
	Bone markers (NTX,DPD, Ca,s-BAP)	0.78	0.31	0.14	0.001	0.59	0.11	0.12	0.001
	Vitamin D and Ca intake	0.48	0.21	0.15	0.001	0.31	0.10	0.11	0.01
	physical activity/sun exposure	0.57	0.36	0.28	0.001	0.48	0.21	0.16	0.01
	Adiposity (BMI, WHtR)	0.66	0.31	0.42	0.001	0.42	0.22	0.28	0.01
BSI	Hand grip strength(left/right)	0.75	0.64	0.34	0.001	0.46	0.23	0.19	0.001
	Vitamin 25(OH)D status	0.65	0.32	0.26	0.001	0.39	0.18	0.20	0.001
	Bone markers (NTX,DPD, Ca,s-BAP)	0.71	0.38	0.31	0.001	0.64	0.21	0.25	0.001
	Vitamin Dand Ca intake	0.53	0.25	0.15	0.001	0.41	0.16	0.10	0.01
	physical activity/sun exposure	0.54	0.26	0.25	0.001	0.38	0.17	0.18	0.01
	Adiposity (BMI, WHtR)	0.61	0.35	0.29	0.001	0.48	0.19	0.21	0.01
Osteioprosis	Hand grip strength(left/right)	0.56	0.48	0.16	0.001	0.36	0.31	0.11	0.001
	Vitamin 25(OH)D status	0.68	0.32	0.21	0.001	0.46	0.16	0.09	0.001
	Bone markers (NTX,DPD, Ca,s-BAP)	0.74	0.46	0.25	0.001	0.58	0.21	0.13	0.001
	Vitamin D and Ca intake	0.72	0.43	0.22	0.01	0.59	0.19	0.17	0.001
	physical activity/sun exposure	0.59	0.26	0.29	0.01	0.38	0.15	0.21	0.01
	Adiposity (BMI, WHtR)	0.58	0.28	0.26	0.01	0.49	0.21	0.12	0.01

*BMD* Mineral bone density, *BMC* Bone mineral content, *BSI* Bone stiffness index, *SEE* Standard Error of the Estimate, *BMI* Body mass index, *WHtR* Waist to height ratio, *PA* Physical activity, *Ca* Calcium, *sBAP* Serum bone-specific alkaline phosphatase, *DPD (nM/MCl)* Deoxypyridinoline, *NTX (nM BCE/mM Cr)* Cross-linked N-telopeptides of type I collagen

## Discussion

The results of our current research significantly illustrate that the HGS, vitamin D status, physical activity, diets, adiposity, and exposure to sunlight are associated moderately with BMD, BSI, BMC, and osteoporosis in school children of both genders.

These results refer to a positive association between bone formation and hand grip strength in the arms. Therefore, in boys, the HGS explains around 48% of the BMD, 69% of the BMC, 64% of the BSI, and 21% of the bone loss compared to girls who showed 29% of the BMD and 26% of the BMC, 23% of the BSI, and 31% of the bone loss respectively. Also, osteoporosis was significantly predicted in 19.64% of the total population most of them were girls (14.3% vs 5.4% for boys; *P* = 0.01). The incidence of osteoporosis was higher and significantly correlated in girls with lower and moderate HGS than in boys of the same category. Several studies showed positive correlations between parameters of bone health and HGS in physically active and non-active subjects [14, 60, 87–90]. These results revealed that the HGS independently could predict the bone health of boys and girls. These systematic associations suggest that elevated levels of HGS help in promoting better bone health by producing a mediating effect over all the musculoskeletal and respiratory systems [14, 90]. HGS showed to be associated with bone mineral density in adolescent students. Lower HGS values significantly correlated with poor scores of the total body BMD and BMC respectively [54]. The defects in hand grip strength were diagnosed as bone fragility in the total body that could be associated with the loss of physical function and a negative impact on recovering health, particularly after an illness or surgery [91, 92]. Our findings, could explain that hand grip isometric exercise provides a promising adaptation to bones via static and dynamic forces created by muscular contractions [93]. Also, bone density and bone minerals content showed to be associated with hand grip strength in children and adolescents [25–28]. Hand grip strength in association with other physiological factors such as maximum oxygen consumption, and maximum expiratory flow showed to be linked with bone mineral density in children, adolescents, and adults [25–28].

In this study, there was adifference in isometric strength between both genders. This could be explained by a greater physical performance level in boys compared to girls. During the same stage of isometric strength, boys are liable to have more performance levels than girls [3]. Additionally, girls may be at a greater risk of evolving bone fragility during adulthood as compared to boys of the same category [94].

In this current study, vitamin D status, and diets containing Ca and vitamin D were shown to be the most promising outcome measures significantly associated with both the scores of HGS and bone health among school children. Lower vitamin 25(OH)D status, and inadequate Ca and vitamin D intake were shown to be significantly associated with lower HGS, bone density markers; BMD, BMC, and BSI, in addition to higher expression values of NTX, DPD, s-BAP, and decline in serum calcium, esecially in girls than boys. This was confirmed by the higher incidence of osteoporosis (19.64%) of the total population studied, most of them were girls (14.3% vs 5.4% for boys; *P* = 0.01).

Our findings are in line with other studies which confirmed the role of calcium and vitamin D intake and vitamin D deficiency, along with other important factors such as genetics, hormonal, physical activity, and adiposity on bone health among children, and adolescents [23, 24].

In children and adults, muscle weakness and “poor bone health were shown to be linked with vitamin D deficiency, this established the indirect benifits role of vitamin D on skeletal health via regulation of calcium homeostasis [43]. The role of vitamin D was clearly observed in muscle function [44], calcium absorption, and maintaining homeostasis [45], and any deficiency in vitamin D produces poor muscle function which appears before the biochemical signs of bone disease [46, 47]. Recently, in schoolchildren, a significant positive association was reported between serum 25(OH)D levels and handgrip strength.

The data of this study clearly imply the importance of vitamin D-rich foods along with calcium intake, and exposure to sunlight in the production of sufficient vitamin D and improving grip strength and bone health among school children [48]. The data also showed that obesity, a sedentary lifestyle or lower PA, and inadequate exposure to sunlight, were significantly considered as related risk factors to bone loss among school children particularly in girls.

In girls, lower values of total bone density parameters; BMD, BMC, BSI, and abnormal expression of biomarkers of bone metabolism; NTC, DPD, Ca, and s-BAP were significantly associated with poor PA, obesity, and inadequate sun exposure. These parameters confer an additional confirmation on the relative higher osteoporosis among girls. Our results are matched with those who reported that physical activity (PA) with higher intensities significantly optimizes skeletal development and significantly aids in preventing age-related bone loss and osteoporotic fractures during childhood [30–33]. This correlation obtained between muscle optimization and bone status was suggested to be mechanical in origin, whereas the skeletal system clearly adapts to both stress and mechanical loads on muscles, producing powerful loading forces on the bone [34]. Also, in school-based interventions, high-impact PA was clearly observed to improve both the muscle force and strength acting on the bone [35, 36]. These observations obviously suggested the pivotal role of muscle strength and muscle mass in bone development during growth [37]. Conversely, children with sedentary life behaviors, such as sitting for a long time in front of screens, watching television, or playing computer games, may have a negative or adverse effect on bone health [31, 38–41].

In general, hand grip strength, vitamin D status, adiposity, PA, sun exposure, and diets containing adequate amounts of Ca and Vitamin D are associated with a certain adverse effects on bones or could predict bone health in school children in both genders [30–33, 48, 87, 95–100].

## Conclusions

The HGS, vitamin D status, PA, diets, adiposity, and sun exposure are positively associated with the bone health of school children aged 6–12 years old of both genders. The students with the lowest hand grip strength and vitamin D values showed lower BMD, BMC, and BSI values. Moreover, it is important to point out that the influence of vitamin D values, PA, diets, adiposity, and sun exposure is greater with regard to bone health in correlation with isometric grip strength in both arms of children. These results strengthen the enclosure of physical exercise, diets, vitamin D, and sunlight exposure to improve skeletal muscle strength and bone health in schoolchildren. In addition, these outcome measures might be considered diagnostic non-invasive predictors of bone health for clinical use in epidemiological contexts; however, more studies are required.

## Data Availability

All data generated or analyzed during this study are presented in the manuscript. Please contact the corresponding author for access to the data presented in this study.
